# Using NMR in saliva to identify possible biomarkers of glioblastoma and chronic periodontitis

**DOI:** 10.1371/journal.pone.0188710

**Published:** 2018-02-06

**Authors:** Alberto García-Villaescusa, José Manuel Morales-Tatay, Daniel Monleón-Salvadó, José Manuel González-Darder, Carlos Bellot-Arcis, José María Montiel-Company, José Manuel Almerich-Silla

**Affiliations:** 1 Departament d’Estomatologia, Facultad de Medicina y Odontología, Universitat de València, Valencia, Spain; 2 Unidad Central de Investigación en Medicina, Facultad de Medicina y Odontología, Universitat de València, Valencia, Spain; 3 Fundación de Investigación del Hospital Clínico Universitario de Valencia, Instituto de Investigación Sanitaria Clínico Valencia (INCLIVA),Valencia, Spain; 4 Department of Neurosurgery, Clinic Hospital, Valencia, Spain; University of Insubria, ITALY

## Abstract

Nowadays there is increasing interest in identifying–and using–metabolites that can be employed as biomarkers for diagnosing, treating and monitoring diseases. Saliva and NMR have been widely used for this purpose as they are fast and inexpensive methods. This case-control study aimed to find biomarkers that could be related to glioblastoma (GBL) and periodontal disease (PD) and studied a possible association between GBL and periodontal status. The participants numbered 130, of whom 10 were diagnosed with GBL and were assigned to the cases group, while the remaining 120 did not present any pathology and were assigned to the control group. On one hand, significantly increased (p < 0.05) metabolites were found in GBL group: leucine, valine, isoleucine, propionate, alanine, acetate, ethanolamine and sucrose. Moreover, a good tendency to separation between the two groups was observed on the scatterplot of the NMR. On the other hand, the distribution of the groups attending to the periodontal status was very similar and we didn´t find any association between GBL and periodontal status (Chi-Square 0.1968, p = 0.91). Subsequently, the sample as a whole (130 individuals) was divided into three groups by periodontal status in order to identify biomarkers for PD. Group 1 was composed of periodontally healthy individuals, group 2 had gingivitis or early periodontitis and group 3 had moderate to advanced periodontitis. On comparing periodontal status, a significant increase (p < 0.05) in certain metabolites was observed. These findings along with previous reports suggest that these could be used as biomarkers of a PD: caproate, isocaproate+butyrate, isovalerate, isopropanol+methanol, 4 aminobutyrate, choline, sucrose, sucrose-glucose-lysine, lactate-proline, lactate and proline. The scatter plot showed a good tendency to wards separation between group 1 and 3.

## Introduction

NMR spectroscopy analysis provides information on both the structure and the composition of low-molecular-mass metabolites in biological fluids and is a rapid and low-cost technique for exploring pathological metabolic processes. The major advantages of NMR spectroscopy include its unbiased metabolite detection, quantitative nature, and high reproducibility. NMR-based metabolomics could be appropriate as a cost-effective solution for high throughput analysis [1]. Saliva is a complex biofluid vital for the maintenance of healthy oral tissues. Saliva is composed predominantly by water but also consists of a mixture of proteins, enzymes, mucus, hormones, electrolytes, ions and small molecular weight. Compared to other biofluids, the saliva collection method is easy, fast and non-invasive [2]. In the last years, screening saliva has been proposed for diagnosing, monitoring and treating several diseases [3].

Glioblastoma (GBL) (2016 WHO classification of CNS tumors) is one of the most lethal primary malignant tumors of the central nervous system, as the mean survival is under 15 months and the five-year rate is under 10%. The risk factors for GBL are unknown, although constant exposure to ionizing radiation or chemical agents can increase its development. GBL is mainly diagnosed at advanced ages, with a mean age on diagnosis of 64 years [4], and has an incidence of 2–3 cases/100.000 inhabitants/year [5]. To improve the life expectancy and quality of life of these patients, an increasing interest in studying the relationship between GBL and other systemic disorders that alter the immune response has been seen in recent years.

Chronic periodontitis (CP) is an inflammatory disorder characterized by the progressive and irreversible destruction of the tissues surrounding the tooth. It affects approximately 50% of the adult population and its incidence and severity increase with age, reaching a prevalence of 70% of over-65 years-old in the USA [6]. Advanced periodontitis is the greatest cause of tooth loss and is therefore a major public health problem. In recent years its relationship with cardiovascular diseases [7], diabetes mellitus [8], and problems during pregnancy [9], has been amply demonstrated. It is also strongly associated with other conditions such as rheumatoid arthritis, chronic obstructive pulmonary disease, pneumonia, obesity, chronic kidney disease, metabolic syndrome and cancer [10]. People with chronic inflammatory diseases of this type may be at greater risk of cancer [11]. Periodontal disorders have been related principally to orodigestive tumors [12], but several studies have observed a relationship between periodontal disease (PD) and the risk of suffering cancers of any type [13]. Controversial differences between the association of systemic diseases with periodontal diseases in differents studies can be used to identify periodontitis due to the heterogeneity in the definitions. No previous studies have specifically addressed the possible relationship between PD and GBL. However, in recent years PD and GBL have been related with an increased activity of citomegalovirus [14,15], making us consider a possible relationship between both. This increased activity of citomegalovirus has been also observed in others inflammatory diseases like rheumatoid arthritis, diabetes mellitus [15], and cardiovascular disease [16].

The aim of this study was to use ^**1**^H NMR to identify whether saliva contained greater concentrations of any particular metabolites which could serve as biomarkers for diagnosing and monitoring GBL and PD and to study a posible association between both diseases.

## Experimental

This case-control study was approved by the Ethics Committee of the Hospital Clínico Universitario of Valencia, Spain (approval number 2012/493) in accordance with the Declaration of Helsinki of 1964 and subsequent amendments by the World Medical Association. The case group comprised hospitalized patients with brain tumors awaiting surgery in the neurosurgery unit of this hospital. Following the operation, the suspected diagnosis was confirmed. The control group was composed of patients from the University of Valencia dental clinic. Oral examination and saliva sampling were performed in both groups.

All the participants of legal age (18 years or over) were given an informed consent document with all the information concerning the study, to be signed voluntarily before taking part. For the control group, those who had taken antibiotics in the past six months, had fewer than eight teeth (excluding third molars), were pregnant or, in general, presented any condition that could lead to error like cardiovascular diseases, diabetes mellitus, rheumatoid arthritis, chronic obstructive pulmonary disease, pneumonia, chronic kidney disease, metabolic syndrome, obesity and Alzheimer´s disease, were excluded from the study [17]. The case group included all patients with a diagnosis of glioblastoma who met the requirement of having at least eight teeth. An oral examination examination was carried out by a single expert examiner following intraexaminer calibration [18]. The participants’ personal details were taken and a full oral examination was performed, recording their periodontal status in greater detail. For this purpose, the probing depths around the two teeth in each quadrant with the greatest probing depth were measured with a Cp12 probe (hu-Friedy, Chicago, IL) at six points: mesio-labial, mid-labial, disto-labial, mesio-palatal/lingual, mid-palatal/lingual and disto-palatal/lingual. The probing depth at each point was recorded, as was the loss of periodontal insertion. The periodontal disease classification system of the American Academy of Periodontology was used to classify the periodontal status of the patients [6]. Following this classification, the participants were divided into three groups in order to obtain results with greater discriminatory power. Group 1 included all the periodontally healthy participants, group 2 was composed of those with gingivitis or early periodontitis and group 3 comprised those with moderate to advanced periodontitis. Age and gender were also recorded.

### Sample collection and preparation

Saliva samples were obtained in the early morning to avoid the introduction of exogenous agents into the oral samples. The participants had not ingested any food, or chewed gum, or brushed their teeth or used any oral hygiene product in the two hours before the sample was taken, and had not smoken for at least one hour before. To collect the saliva samples, we used the “draining method”: the participants were seated comfortably for a few minutes in a resting position with their heads tilted slightly forward, in a quiet environment to avoid non-test stimuli. The slightly parted lips allowed the saliva to fall into a wide-necked sterile container. The liquid collected was then transferred with a pipette to a sterile 1.5 mL Eppendorf tube and was frozen immediately at -80°C until the NMR measurements were made [19,20].

### NMR data acquisition and processing

The data acquisition and processing were conducted as previously described by Galbis-Estrada et al. [21]. Forty-eight microliters of sodium-3´-trimethylsilyl propionate-2,2,3,3-d4 (TSP, 32 mM) in deuterium oxide were mixed with 452 μl of saliva and placed in a 5mm high-resolution NMR tube. NMR spectra were acquired using a standard one-dimensional pulse sequence with water suppression in a Bruker Avance DRX 600MHz spectrometer (Bruker Biospin GmbH, Rheinstetten, Germany). A total of 256 FIDs (free induction decay signals) were collected into 32k data points with a spectral width of 14 ppm at 298K. Water pre-saturation for 1 s during the recycling delay was used for solvent signal suppression. Before Fourier transformation, the FID was multiplied by 0.3 Hz of exponential line broadening. All the spectra were phased, the baseline was corrected carefully, and chemical shifts were adjusted with reference to the TSP signal using MestReNova 10 software (Mestrelab Research S.L., Santiago de Compostela, Spain). The spectra were binned into 0.005 ppm buckets between 0.5–10 ppm and the mean was centered for multivariate analysis and normalized to the total aliphatic spectral area (0.5–4.5 ppm) to eliminate differences in total concentrations of metabolites. The data were imported into MATLAB R2013a (The MathWorks Inc., Natick, MA 2013) for additional processing and further analysis. The signals of selected metabolites were integrated and quantified using semi-automated in-house MATLAB peak-fitting routines. Resonances were assigned with reference to the previous literature [20] and to databases: The Human Metabolome Database (http://www.hmdb.ca) and the Chenomx spectral database contained in Chenomix NMR Suite 8.1 (Chenomx Inc, Alberta, Canada).

### Statistical analysis

Chemometrics statistical analyses were performed using in-house MATLAB scripts and the PLS Toolbox 6.7 (Eigenvector Research, Inc., Wenatchee, WA, USA). Metabolite levels were computed from the raw (untransformed) data and expressed as mean ± SD (standard deviation). T-Student’s test was used to determine the statistical significance of differences between the means in both of the cases, and the control group and ANOVA to estimate the differences between the three categories of periodontal status. A chi- squared was used for comparative proportions. The significance level was p < 0.05. Principal component analysis (PCA) and projection to latent structures for discriminant analysis (PLS-DA) were applied to the NMR spectral datasets. Results were cross-validated using the leave-one-out to evaluate the accuracy of each classification model [22]; in each run one sample of the data is left out of the training and used to test the model. The whole cross validation process was run 10 times. The results of the cross validation were evaluated by the Q2 parameter. Q2 is the averaged correlation coefficient between the dependent variable and the PLS-DA predictions and provides a measure of prediction accuracy during the cross-validation process (higher values mean better prediction).

## Results

Ten participants met the requirements for assigning the case group. This was performed by 1 male (10%) and 9 females (90%) in which the average age was 54.7 years old, range 26–78. In contrast, the control group comprised 120 participants of whom 49 were males (40.8%) and 71 were females (59.2%,). The average age was 51.8 years old, range 19–81 (Table 1).

**Table 1:** Distribution of case and control groups.

	Case group	Control group
Participants (n)	10	120
Gender	1 male (10%)/ 9 female (90%)	49 male (40.8%) 71 female (59.2%)
Age	54.7 years old (26–78)	Age: 51.8 years old (19–81)

**Fig 1:**
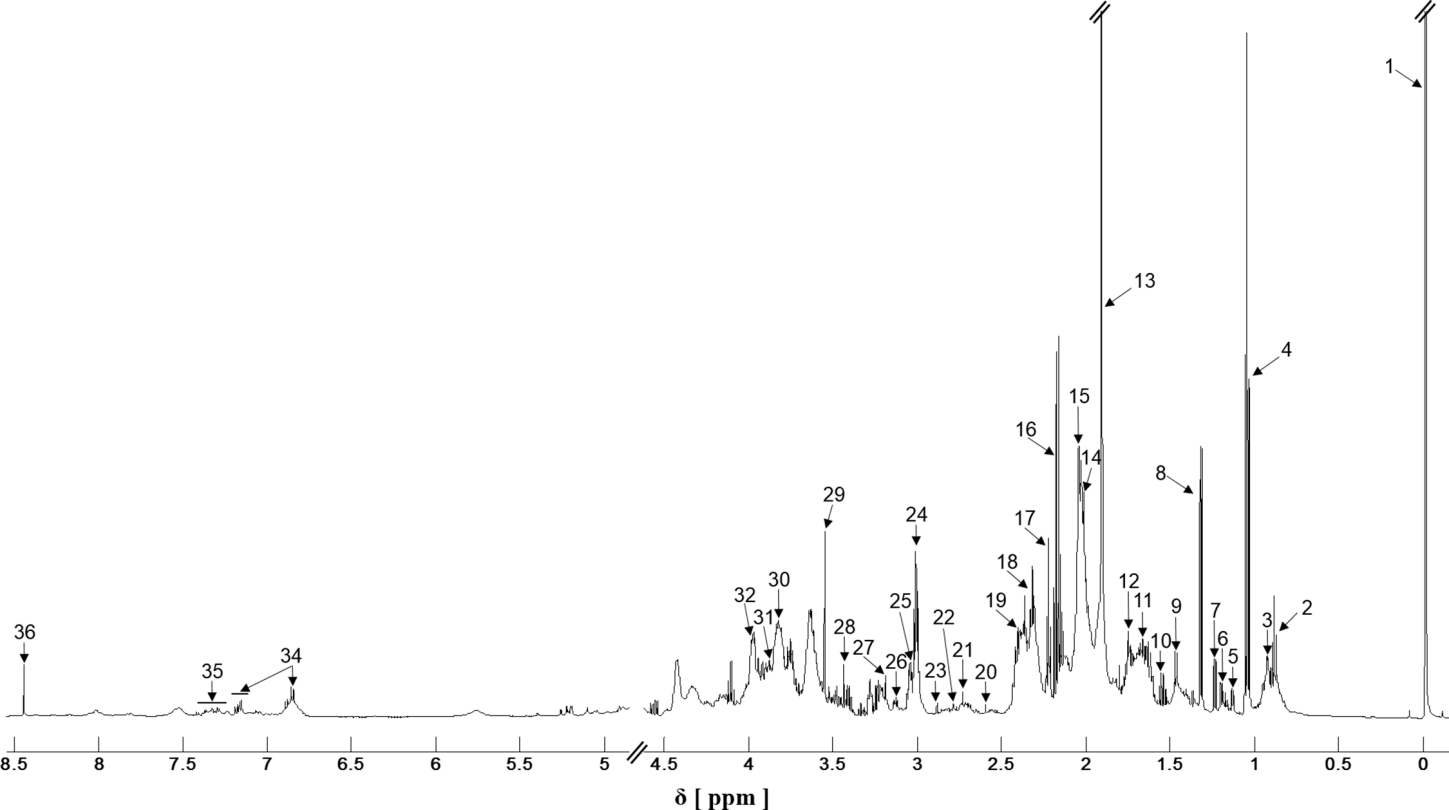
A typical H NMR spectrum of saliva. 1. TSP 2. Isocaprate+butyrate 3. Leucine+isoleucine 4. Propionate 5. Propylene glycol 6. Ethanol 7. Fucose 8. Lactate 9. Alanine 10. Butyrate 11. 2-aminoadipate 12. Leucine 13. Acetate 14. Proline 15. Glutamate+isovalerate 16. Propionate 17. 2-aminoadipate 18. Proline+Glutamate 19. Succinate 20. Methylamine 21. Sarcosine 22. Aspartate 23. Trimetylamine 24. 4Aminobutyrate+Lysine 25. Ornithine 26. Phenylalanine 27. Choline 28. Proline 29. Glycine 30. Sucrose 31. Glucose 32. Phenylalanine 33. Lactate 34. Tyrosine 35. Phenylalanine 36. Formate.

The identification and quantification of the saliva metabolite concentrations were made using the Human Metabolome Database (http://www.hmdb.ca) and the Chenomx spectral database contained in Chenomix NMR Suite 8.1 sofware (Chenomx Inc, Alberta, Canada). The databases compare metabolites together with their respective concentrations based on a known reference signal, in this study we used TSP, 32 mM. A sample ^**1**^H NMR spectrum of saliva from a participant is shown in Fig 1. The spectra of saliva were dominated by acetate, propionate and TSP. It is worth remembering that TSP was contained in the solution used to NMR processing.

**Fig 2:**
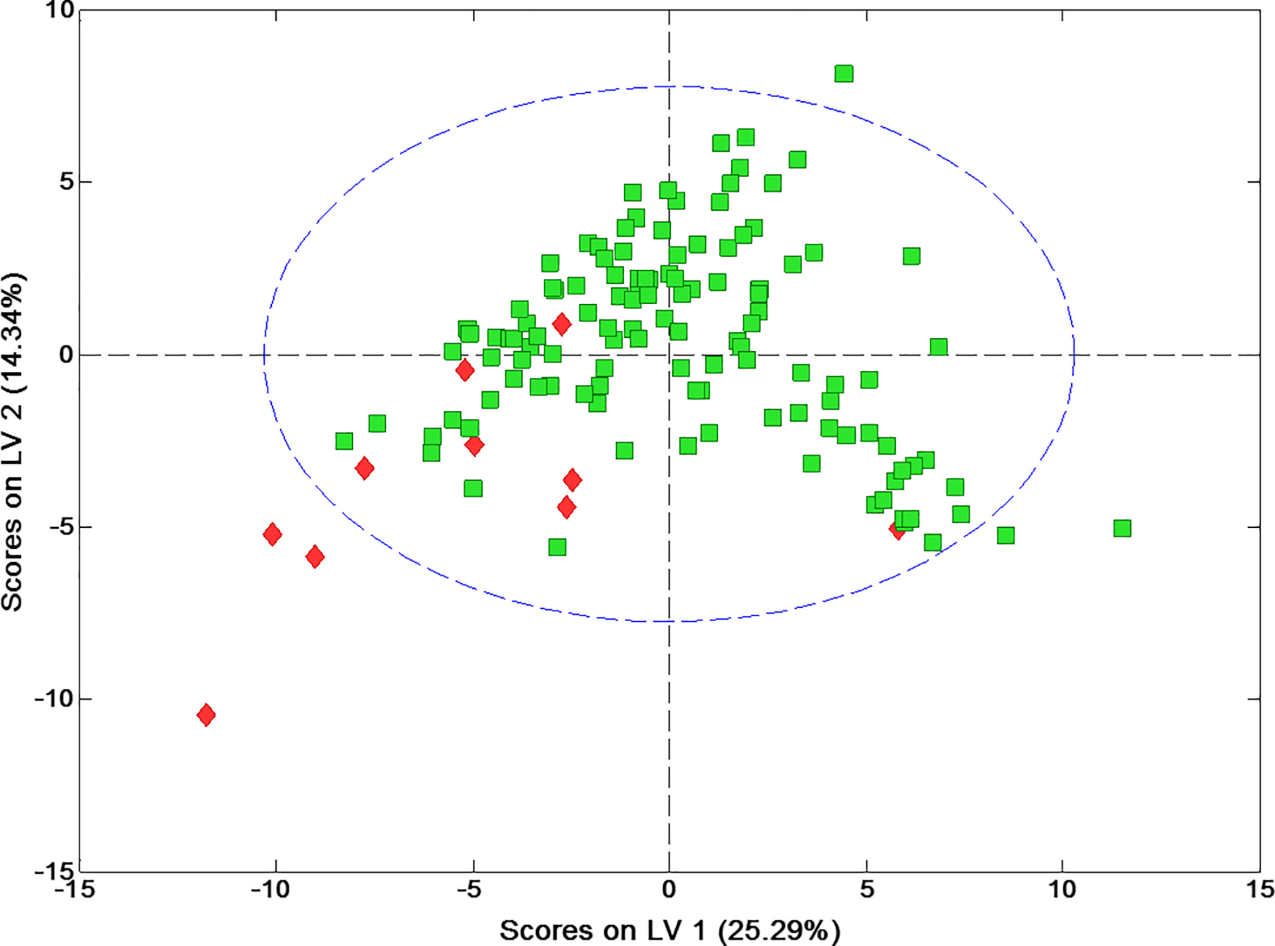
PLS-DA scores to differentiate between patients with GBL (red) and without GBL (green).

A total of 68 metabolites were assigned, quantified and included at the statistical test (S1 Table). On comparing means between case and control groups, the subjects with GBL were found to have significantly increased (p < 0.05) saliva concentrations of particular metabolites: leucine, valine, isoleucine, propionate, alanine, acetate and ethanolamine. Concentrations of propionate and sucrose were very significant, p < 0.01 (Table 2). The PCA did not show a spontaneous grouping of samples according to the classification into control and GBL patients, so PLS-DA was used to maximize the separation of the two groups and also to reveal specific metabolic changes in the defined groups which enhance the separation between the samples from these groups. Firstly, the two PLS components which explain 39.6% of the total variation were used to build the model, to remove the data variations unrelated to class information. The results are presented as a scatter plot (Fig 2), in which a good tendency to separation between the control and glioblastoma group was observed.

**Table 2:** Metabolites identified and compared between GBM and control groups. * p < 0.05 and ** p < 0.01.

	GBL n = 10		Healthy n = 120	
	Mean	Standar deviation	Mean	Standar deviation	p
Leucine*	.00446934	.001225769	.00571786	.001750212	0.029
Valine*	.00289361	.001258720	.00384140	.001431770	0.045
Isoleucine*	.00262944	.001269018	.00357674	.001359431	0.035
Propionate**	.02450194	.013050096	.01557078	.008892744	0.004
Alanine*	.00681912	.001431254	.00810623	.001792885	0.029
Acetate*	.10065490	.063830358	.06675881	.039332489	0.014
Propionate**	.01880233	.007052425	.01249370	.004660907	0.0001
Ethanolamine*	.00172565	.000598114	.00225825	.000701277	0.021
Sucrose**	.00419344	.000828839	.00547925	.001353303	0.004

The distribution of the groups attending the periodontal status were very similar (Table 3) and we didn´t find asociation between GBL and periodontal status (Chi-Square 0.1968, p = 0.91).

**Table 3:** Distribution of case and control groups attending to the periodontal status.

	Case group n = 10	Control group n = 120
Periodontally healthy	3 (30%)	36 (30%)
Gingivitis/early periodontitis	4 (40%)	55 (45.8%)
Moderate/Advanced periodontitis	3 (30%)	29 (24.2%)

**Fig 3:**
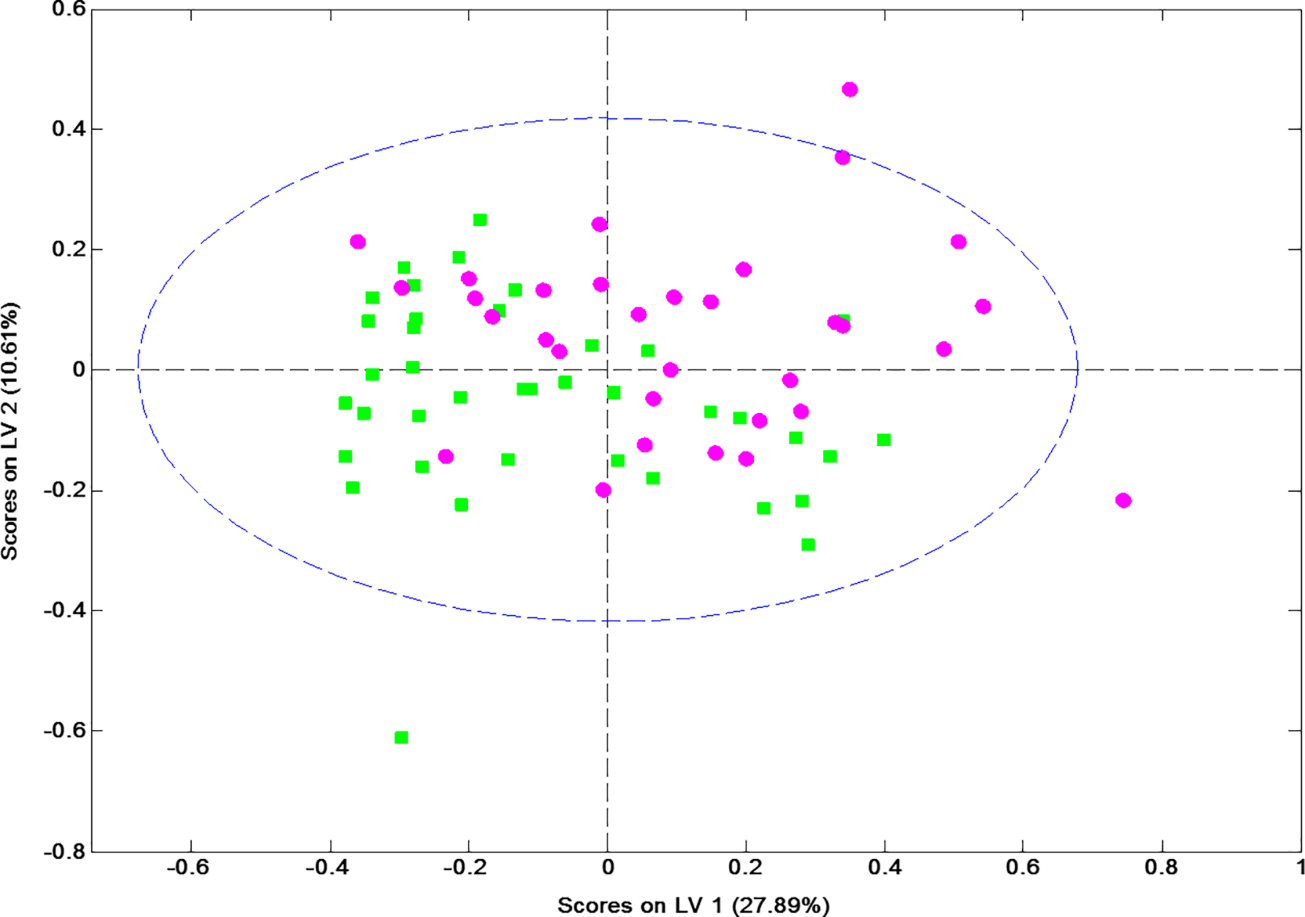
PLS-DA scores of participants with moderate/advanced periodontitis (purple, group 3) and without periodontal problems (green, group 1).

Considering the periodontal status of the sample as a whole (130 participants), a total of 39 subjects were placed in group 1 (periodontally healthy), 59 in group 2 (gingivitis/early periodontitis) and 32 in group 3 (moderate/advanced periodontitis). A total of 68 metabolites were assigned, quantified and included at an ANOVA test (S2 Table). On comparing groups, the subjects with PD were found to have significantly increased saliva concentrations of particular metabolites (p < 0.05): caproate, isocaproate+butyrate, isovalerate, isoleucine, isopropanol+methanol, 4 aminobutyrate, choline, sucrose, sucrose-glucose-lysine, lactate-proline, lactate and proline. Moreover, caproate, isocaproate+butyrate, isovalerate, lactate-proline and proline were very significant, p < 0.01 (Table 4). Additionally, based on these differences in metabolite levels, a model was constructed to discriminate between the two most opposite group (1 Vs 3). In the resulting multivariate space, the two subgroups presented overlaps but a good tendency to separation was observed (Fig 3). The model’s goodness-of-fit metrics were Q2 = 0.119, R2Y = 0.717, and the area under the ROC curve as 0.69.

**Table 4:** Metabolites identified and compared attending the periodontal satatus. * p < 0.05 and ** p < 0.01.

	Periodontal status						
	Periodontally healthy		Gingivitis/early periodontitis		Moderate/Advanced periodontitis		
	Mean	Standar deviation	Mean	Standar deviation	Mean	Standar deviation	p-value
Caproate**	.005886	.001438	.005855	.001990	.006261	.002163	.00531
Isocaprate+butyrate**	.008794	.001343	.009382	.002751	.010995	.003623	.00005
Isovalerate**	.005233	.001176	.005702	.001588	.006760	.002470	.00047
Isoleucine*	.008548	.001283	.008509	.002108	.009497	.002249	.01187
Isopropanol+Methanol*	.010647	.009113	.009472	.006538	.012522	.018816	.04489
4-Aminobutyrate*	.003381	.000825	.003484	.000939	.004105	.001216	.01407
Choline*	.002397	.000431	.002379	.000429	.002444	.000597	.02388
Sucrose*	.005860	.001630	.005397	.001031	.004765	.001339	.02564
Sucrose+Glc+Lys*	.025448	.009300	.024012	.006007	.021597	.004739	.04969
Lactate+Proline**	.011513	.002817	.011886	.005237	.010010	.002031	.00536
Lactate*	.002194	.000798	.002237	.001264	.001796	.000570	.02092
Proline **	.001810	.000527	.001946	.000898	.001653	.000415	.00934

## Discussion

Numerous metabolites have been proposed for diagnosing, monitoring and treating inflammatory diseases. Identification and quatification leves of N-acetyl aspartate (NAA), choline, glutamate, glutamine, lactate, alanine, glucose, inositol, creatinine and lipids have been useful in previous studies to separate glial tumors by type and grade, and determine the choice of therapy and treatment efficacy to evaluate the progression or remission of GBL [23–25]. The present study used NMR to identify biomarkers present in the saliva of both GBL and PD patients. We found significant increases (p < 0.05) of several metabolites between the case and control: leucine, valine, isoleucine, propionate, alanine, acetate, ethanolamine and sucrose. Leucine, valine, isoleucine, alanine, ethanolamine and sucrose was more concentrated in control patients, whereas pronionate and acetate were more concentrated in GBL patients. According to our results, all these metabolites could be useful on monitoring GBL but particularly important could be sucrose and propionate as they were very significantly increased (p < 0.01). This is the first study that uses HNMR in saliva samples to identify possible biomarkers of GBL but we have found some metabolites significantly increased in GBL patients in accordance with previous studies that uses tumor cells [26, 27]. We didn´t find asociation between GBL and periodontal status (Chi-Square 0.1968, p = 0.91). Subsequently, the sample as a whole (130 individuals) was divided into three groups by periodontal status in order to identify biomarkers for PD. Group 1 was composed of periodontally healthy individuals, group 2 had gingivitis or early periodontitis and group 3 had moderate to advanced periodontitis.We also find significant increases in particular metabolites concentrations in PD patients, as were also found in previous studies [28, 29]: Caproate, isoleucine, isopropanol-methanol and choline concentrations were reduced on gingivitis/eartly periodontitis but increased in periodontitis moderate/severe with respect to those periodontally healthy. Isocaproate+butyrate, isovalerate and 4-aminobutyrate concentrations were increased as the periodontal stadium worsened while sucrose and sucrose-glucose-lysine concetrations were reduced. Finally, concentrations of lactate-proline, lactate and proline, were increased in group 2 but decreased in group 3 with respect to the group 1. According to our results, all these metabolites could be useful for diagnosing and treating PD, but particularly important could be caproate, isocaproate-butyrate, isovalerate, lacatate+proline and proline, as they were very significantly increased (p < 0.01).

Short chain fatty acids such as butyrate, caproate, isocaproate, propionate, isovalerate and lactate play an important role in periodontal disorders. They are end-products of bacterial metabolism and have been strongly linked to deep periodontal pockets, loss of insertion, bleeding and inflammation. The acids prevent cell division, making repair difficult and favoring junctional epithelium degeneration processes, which in turn allows the entry of pathogens and the formation of periodontal pockets. These bacterial metabolites stimulate an inflammatory response and the liberation of cytokines. At cell level, they inhibit leukocyte apoptosis and cell proliferation in the gingival epithelium and endothelium, preventing their repair. [30]. As has been shown, these metabolites diminish significantly following periodontal treatment and gradually increase over time, so they have been pointed to as possible indicators of PD development and progression [31]. Isopropanol+metanol was augmented is associated with moderately increased severity of periodontal disease [32]. Propionate levels can rise as there is a significant increase in alpha-amino-3-hydroxy-5-methylisoxazole-4-propionic acid (AMPA) receptors, which can play an important biological role in tumor invasion [33]. A significant rise in sucrose-glucose-lysine can modify receptors on the cell surface, encouraging activation of the immune response initiated by the cytokines, which, as in diabetes, can provoke an exacerbated inflammatory response to pathogenic bacteria present in the gum, and also modifies the capacity for resolution of inflammation and subsequent repair, accelerating the destruction of periodontal support tissues [34]. Equally, there is increasing evidence that blood glucose levels and glucose metabolism play an important role in the development and growth of cancers, including glioblastomas [35]. Alanine has been suggested as a possible biomarker for predicting the progression of periodontal disease by measuring alanine transferase (ALT) enzyme levels in saliva [36]. It also rises in malignant brain tumors and can therefore be used to distinguish the type of tumor and degree of malignity [37]. Increased proline levels can be due to destruction of the periodontal ligament by bacteria, liberating the amino acids that constitute the collagen in this ligament’s fibers. Also, proline liberation as part of the host’s inflammatory response seeks to prevent new bacteria from attaching themselves to the surface of the host. Diseases that cause tissue damage liberate different enzymes related to cell death and destruction, such as lactate dehydrogenase (LDH), which would explain the increased lactate levels in individuals with moderate to advanced periodontitis. LDH is a specific indicator for periodontal lesions and a significant association with deep periodontal pockets has been found [38]. This enzyme is very useful for checking on PD activity or assessing the effect of periodontal treatments, and can also be helpful for monitoring PD progression [39]. It has also been suggested for diagnosing and monitoring glioblastoma [40] but in our study we didn´t find such an increase in GBL patients. Leucine-rich proteins have been related to the capacity to mediate in bacterial adhesion and invasion of gingival epithelium cells, fibroblasts and endothelial cells [41]. Furthermore, an increase in leucine-rich proteins has been observed in some malignant gliomas and has been related principally to a greater risk of suffering astrocytomas and glioblastomas [42].

## Conclusion

The present study found significant differences in some metabolites in saliva of GBL and PD patients but any association between GBM and periodontal status was found. Sucrose and propionate in GBL patients and caproate, isocaproate-butyrate, isovalerate, lacatate+proline and proline in PD patients, were very significantly increased (p < 0,01). Previous studies have analyzed saliva using ^**1**^H NMR or LS-MS to identify metabolites related with PD, collect and analyze saliva samples seems to be a good and useful method to follow-up different diseases due to the fact that it is an easy, fast and non-invasive option, and its results are similar to those obtained from serum analysis or tissue cells cultures. There are a lack of studies in screening saliva to find biomarkers of GBL. In this study, we used ^**1**^H NMR but other complementary analytical platforms from the same sample, as such Mass Spectrometry, would be necessary to conclude if saliva could be used in the screening of GBL.

## Supporting information

S1 Table Metabolites identified and compared between GBL and control groups. The statistically significant ones are marked * p < 0.05 and ** p < 0.01.
(DOCX)

S2 Table Metabolites identified and compared attending the periodontal satatus. The statistically significant ones are marked * p < 0.05 and ** p < 0.01.
(DOCX)

## Abbreviations

CP: Chronic periodontitis
GBL: Glioblastoma
PD: Periodontal disease
