# Supplementary material for: Using NMR in saliva to identify possible biomarkers of glioblastoma and chronic periodontitis
Source: PLoS One. 2018 Feb 6;13(2):e0188710. doi: 10.1371/journal.pone.0188710 (PMC5800567; doi:10.1371/journal.pone.0188710)
Supplement: S1 Table — The statistically significant ones are marked * p < 0.05 and ** p < 0.01. (DOCX) [file pone.0188710.s001.docx]

|  | GBL n = 10 | | Healthy n = 120 | |  |
| --- | --- | --- | --- | --- | --- |
|  | Mean | Standar deviation | Mean | Standar deviation | p |
| Caproate | .00547079 | .001808002 | .00600538 | .001889839 | 0.390 |
| Isocaprate+butyrate | .01100446 | .004068350 | .00948581 | .002648535 | 0.098 |
| Isovalerate | .00616633 | .002358222 | .00579292 | .001784973 | 0.537 |
| Isoleucine | .00871156 | .002732246 | .00876805 | .001907168 | 0.931 |
| Leucine+Isoleucine | .00729936 | .002062886 | .00897039 | .002612769 | 0.051 |
| Leucine* | .00446934 | .001225769 | .00571786 | .001750212 | 0.029 |
| Valine* | .00289361 | .001258720 | .00384140 | .001431770 | 0.045 |
| Isoleucine* | .00262944 | .001269018 | .00357674 | .001359431 | 0.035 |
| Valine | .00118553 | .000528283 | .00144191 | .000577423 | 0.177 |
| Propionate** | .02450194 | .013050096 | .01557078 | .008892744 | 0.004 |
| Propylene glycol | .00276970 | .001308963 | .00346145 | .002019140 | 0.290 |
| Isopropanol | .00078042 | .000315510 | .00100705 | .000730055 | 0.333 |
| Isopropanol+Methanol | .00993557 | .006001652 | .01062841 | .011766043 | 0.855 |
| 3-hydroxybutyrate+fucose | .01365722 | .005984835 | .01440969 | .011987207 | 0.845 |
| Fucose | .00182622 | .000519961 | .00184766 | .000661022 | 0.921 |
| Fucose | .00548051 | .002250512 | .00571742 | .002311854 | 0.756 |
| Lactate | .00946763 | .002857967 | .01204786 | .005004976 | 0.111 |
| Ibuprofen | .00340384 | .001352378 | .00371495 | .001420107 | 0.505 |
| Alanine* | .00681912 | .001431254 | .00810623 | .001792885 | 0.029 |
| Butyrate 20 | .00677715 | .002655320 | .00723742 | .002596280 | 0.592 |
| 2-Aminoadipate | .01905006 | .006877783 | .01657399 | .004523386 | 0.114 |
| Leucine | .02328470 | .005146337 | .02170980 | .004049947 | 0.250 |
| Acetate* | .10065490 | .063830358 | .06675881 | .039332489 | 0.014 |
| Proline | .03622010 | .010937793 | .03580801 | .010573032 | 0.906 |
| Glutamate+isovalerate+proline | .05869351 | .020236196 | .05611458 | .017750410 | 0.663 |
| Glutamine | .01386411 | .002915212 | .01468374 | .003372193 | 0.458 |
| Butyrate+Propionate | .00624964 | .001359694 | .00476038 | .001164302 | 0.0002 |
| Propionate** | .01880233 | .007052425 | .01249370 | .004660907 | 0.0001 |
| 2-Aminoadipate | .01228867 | .004941988 | .01015503 | .003758640 | 0.095 |
| Proline+Glutamate 30 | .02961050 | .009056249 | .02921854 | .007451018 | 0.875 |
| Succinate | .00500601 | .003187608 | .00432747 | .001783900 | 0.284 |
| Glutamine | .02575876 | .011117696 | .02424261 | .007099691 | 0.538 |
| Citrate | .00259712 | .002672051 | .00302090 | .002734251 | 0.638 |
| Methylamine | .00063755 | .000622054 | .00072892 | .000655947 | 0.672 |
| Aspartate | .00199601 | .002122504 | .00276391 | .002107747 | 0.271 |
| Citrate | .00147769 | .000933958 | .00173880 | .000951657 | 0.405 |
| Dimethylamine | .00134702 | .000759714 | .00126726 | .000596354 | 0.691 |
| Sarcosine | .00152068 | .000789726 | .00170213 | .000784733 | 0.484 |
| Aspartate | .00353876 | .002703533 | .00442336 | .002620869 | 0.308 |
| Trimethylamine 40 | .00107753 | .000901160 | .00128072 | .000950781 | 0.516 |
| 4-Aminobutyrate | .00416788 | .001218745 | .00355912 | .000991018 | 0.069 |
| 4-Aminobutyrate+Lysine | .01275377 | .004246529 | .01133193 | .003785865 | 0.260 |
| Ornithine | .00488256 | .001819615 | .00441179 | .001535929 | 0.360 |
| Phenylalanine | .00324466 | .001566820 | .00393267 | .001566020 | 0.184 |
| Ethanolamine* | .00172565 | .000598114 | .00225825 | .000701277 | 0.021 |
| Choline | .00214230 | .000396780 | .00242211 | .000474055 | 0.072 |
| Glucose+Taurine | .00431735 | .000991375 | .00519765 | .001234927 | 0.030 |
| Taurine | .00093999 | .000562936 | .00119376 | .000587494 | 0.190 |
| Phenylalanine | .00376227 | .002078896 | .00349482 | .002239130 | 0.716 |
| Proline 50 | .00458733 | .003815527 | .00660678 | .003969150 | 0.124 |
| Glucose | .00066679 | .000657870 | .00090781 | .000653960 | 0.265 |
| Tau+Pro+Glc | .00862911 | .003182442 | .01043649 | .003257966 | 0.094 |
| Glycine | .00472351 | .001892298 | .00608500 | .002935841 | 0.153 |
| Sucrose** | .00419344 | .000828839 | .00547925 | .001353303 | 0.004 |
| Sucrose+Glc+Lys | .02246877 | .006679400 | .02396305 | .007051476 | 0.519 |
| Glucose+Sucrose | .03373861 | .008053195 | .03414689 | .008230252 | 0.880 |
| Glucose | .00457122 | .000925483 | .00451373 | .000970737 | 0.857 |
| Tyrosine | .01132537 | .002084099 | .01210857 | .002471972 | 0.333 |
| Glycolate | .00304212 | .000759103 | .00331044 | .000919918 | 0.372 |
| Phenylalanine 60 | .01550078 | .004817830 | .01620784 | .004256211 | 0.618 |
| Lactate+Proline | .01027046 | .003158200 | .01139885 | .004093359 | 0.397 |
| Lactate | .00198522 | .000885877 | .00212644 | .001021146 | 0.672 |
| Proline | .00165134 | .000482549 | .00184800 | .000721871 | 0.400 |
| Glucose | .00083003 | .002278406 | .00258771 | .004921691 | 0.266 |
| Tyrosine | .00330126 | .000895614 | .00466689 | .003123512 | 0.172 |
| Tyrosine | .00255390 | .000992124 | .00381192 | .002936413 | 0.181 |
| Phenylalanine | .00920208 | .004266676 | .01265773 | .010690748 | 0.313 |
| Formate | .00128695 | .000702510 | .00161428 | .001231129 | 0.409 |

S1 Table. Metabolites identified and compared between GBL and control groups. The statistically significant ones are marked * p < 0.05 and ** p < 0.01.
