# Supplementary material for: Using NMR in saliva to identify possible biomarkers of glioblastoma and chronic periodontitis
Source: PLoS One. 2018 Feb 6;13(2):e0188710. doi: 10.1371/journal.pone.0188710 (PMC5800567; doi:10.1371/journal.pone.0188710)
Supplement: S2 Table — The statistically significant ones are marked * p < 0.05 and ** p < 0.01. (DOCX) [file pone.0188710.s002.docx]

|  | Periodontal status | | | | | |  |  |
| --- | --- | --- | --- | --- | --- | --- | --- | --- |
|  | Periodontally healthy n = 39 | | Gingivitis/early periodontitis n=59 | | Moderate/Advanced periodontitis n=32 | |  |  |
|  | Mean | Standar deviation | Mean | Standar deviation | Mean | Standar deviation | p-value |  |
| Caproate** | | .005886 | .001438 | .005855 | .001990 | .006261 | .002163 | .00531 |
| Isocaprate+butyrate** | | .008794 | .001343 | .009382 | .002751 | .010995 | .003623 | .00005 |
| Isovalerate** | | .005233 | .001176 | .005702 | .001588 | .006760 | .002470 | .00047 |
| Isoleucine* | | .008548 | .001283 | .008509 | .002108 | .009497 | .002249 | .01187 |
| Leucine+Isoleucine | | .008291 | .002224 | .008888 | .002647 | .009427 | .002891 | .33670 |
| Leucine | | .005274 | .001473 | .005696 | .001808 | .005909 | .001908 | .38782 |
| Valine | | .003511 | .001316 | .003879 | .001439 | .003879 | .001575 | .79370 |
| Isoleucine | | .003317 | .001286 | .003597 | .001398 | .003560 | .001442 | .74675 |
| Valine | | .001316 | .000568 | .001440 | .000575 | .001518 | .000586 | .82260 |
| Propionate | | .014155 | .009296 | .015657 | .008858 | .019928 | .010192 | .83822 |
| Propylene glycol | | .003460 | .001763 | .003224 | .001772 | .003685 | .002542 | .31754 |
| Isopropanol | | .001020 | .000809 | .000966 | .000534 | .000995 | .000867 | .45826 |
| Isopropanol+Methanol* | | .010647 | .009113 | .009472 | .006538 | .012522 | .018816 | .04489 |
| 3-hydroxybutyrate+fucose | | .014337 | .009031 | .013349 | .007054 | .016219 | .019068 | .06174 |
| Fucose | | .001780 | .000665 | .001914 | .000656 | .001800 | .000626 | .43593 |
| Fucose | | .005490 | .002175 | .005940 | .002510 | .005511 | .002052 | .20448 |
| Lactate | | .012554 | .005013 | .011588 | .005265 | .011473 | .004117 | .65407 |
| Ibuprofen | | .003544 | .001304 | .003628 | .001375 | .003987 | .001598 | .97450 |
| Alanine | | .007563 | .001459 | .008057 | .002008 | .008458 | .001676 | .47127 |
| Butyrate 20 | | .006859 | .002367 | .007152 | .002558 | .007713 | .002907 | .83190 |
| 2-Aminoadipate | | .015970 | .004189 | .016286 | .004747 | .018615 | .005067 | .19893 |
| Leucine | | .021022 | .003503 | .021338 | .004438 | .023725 | .003808 | .59404 |
| Acetate | | .063172 | .041177 | .066075 | .041253 | .082984 | .044056 | .88340 |
| Proline | | .036210 | .009817 | .034946 | .011454 | .037037 | .009855 | .34550 |
| Glutamate+isovalerate+proline | | .056389 | .016826 | .055198 | .019293 | .058276 | .016750 | .38693 |
| Glutamine | | .014215 | .002987 | .014785 | .003762 | .014813 | .002934 | .21540 |
| Butyrate+Propionate | | .004574 | .001312 | .004787 | .001156 | .005403 | .001173 | .79591 |
| Propionate | | .011894 | .005315 | .012740 | .004494 | .014742 | .005699 | .57362 |
| 2-Aminoadipate | | .009674 | .003780 | .010329 | .003814 | .011086 | .004100 | .48738 |
| Proline+Glutamate 30 | | .029163 | .006683 | .028891 | .008307 | .030012 | .007230 | .18424 |
| Succinate | | .004490 | .002085 | .004134 | .001376 | .004698 | .002489 | .56559 |
| Glutamine | | .024307 | .006336 | .023993 | .008008 | .025098 | .007749 | .63493 |
| Citrate | | .002915 | .002823 | .003429 | .002757 | .002265 | .002433 | .24327 |
| Methylamine | | .000711 | .000681 | .000821 | .000658 | .000553 | .000583 | .20779 |
| Aspartate | | .002703 | .002193 | .003005 | .002148 | .002154 | .001878 | .19518 |
| Citrate | | .001772 | .000995 | .001807 | .000964 | .001490 | .000852 | .26447 |
| Dimethylamine | | .001255 | .000617 | .001320 | .000623 | .001211 | .000578 | .46465 |
| Sarcosine | | .001720 | .000800 | .001786 | .000806 | .001470 | .000697 | .20598 |
| Aspartate | | .004306 | .002677 | .004768 | .002692 | .003655 | .002354 | .18746 |
| Trimethylamine 40 | | .001183 | .000977 | .001404 | .000965 | .001108 | .000858 | .34612 |
| 4-Aminobutyrate* | | .003381 | .000825 | .003484 | .000939 | .004105 | .001216 | .01407 |
| 4-Aminobutyrate+Lysine | | .010653 | .003512 | .011241 | .003740 | .012771 | .004108 | .34458 |
| Ornithine | | .004245 | .001634 | .004598 | .001550 | .004419 | .001490 | .78729 |
| Phenylalanine | | .004029 | .001505 | .004116 | .001601 | .003262 | .001472 | .38977 |
| Ethanolamine | | .002317 | .000769 | .002245 | .000619 | .002044 | .000768 | .18408 |
| Choline* | | .002397 | .000431 | .002379 | .000429 | .002444 | .000597 | .02388 |
| Glucose+Taurine | | .005465 | .001242 | .005045 | .001172 | .004878 | .001299 | .88274 |
| Taurine | | .001220 | .000606 | .001198 | .000617 | .001074 | .000510 | .28431 |
| Phenylalanine | | .003460 | .002180 | .003785 | .002336 | .003086 | .002040 | .22045 |
| Proline 50 | | .006419 | .003581 | .007152 | .004241 | .005200 | .003740 | .09392 |
| Glucose | | .000966 | .000609 | .000955 | .000704 | .000675 | .000582 | .05260 |
| Tau+Pro+Glc | | .010910 | .003253 | .010209 | .003404 | .009713 | .003028 | .88484 |
| Glycine | | .005885 | .002211 | .006165 | .003203 | .005757 | .003060 | .23932 |
| Sucrose* | | .005860 | .001630 | .005397 | .001031 | .004765 | .001339 | .02564 |
| Sucrose+Glc+Lys* | | .025448 | .009300 | .024012 | .006007 | .021597 | .004739 | .04969 |
| Glucose+Sucrose | | .035320 | .008926 | .033338 | .007911 | .034080 | .007822 | .58635 |
| Glucose | | .004649 | .001162 | .004485 | .000852 | .004421 | .000905 | .12421 |
| Tyrosine | | .012690 | .002685 | .012245 | .002432 | .010904 | .001748 | .05989 |
| Glycolate | | .003343 | .000924 | .003242 | .000917 | .003313 | .000900 | .85753 |
| Phenylalanine 60 | | .016250 | .004255 | .016090 | .004360 | .016153 | .004319 | .96426 |
| Lactate+Proline** | | .011513 | .002817 | .011886 | .005237 | .010010 | .002031 | .00536 |
| Lactate* | | .002194 | .000798 | .002237 | .001264 | .001796 | .000570 | .02092 |
| Proline ** | | .001810 | .000527 | .001946 | .000898 | .001653 | .000415 | .00934 |
| Glucose | | .002438 | .004516 | .002642 | .005780 | .002122 | .002778 | .16834 |
| Tyrosine | | .004418 | .002037 | .004891 | .003905 | .004130 | .002048 | .32994 |
| Tyrosine | | .003553 | .001684 | .004099 | .003669 | .003206 | .002149 | .32517 |
| Phenylalanine | | .011645 | .006222 | .013813 | .013488 | .010683 | .007223 | .25946 |
| Formate | | .001612 | .000911 | .001637 | .001433 | .001473 | .001057 | .38034 |

S2 Table. Metabolites identified and compared attending the periodontal satatus. The statistically significant ones are marked * p < 0.05 and ** p < 0.01.
